# A survey of attitudes towards the curriculum for clinical medicine postgraduates pursuing professional master’s degrees: perspectives of supervisors and students

**DOI:** 10.3389/fmed.2024.1488139

**Published:** 2024-12-10

**Authors:** Rui Xu, Jing Wu, Xin Jin, Min Tang, Caishuang Pang, Zhu Yang, Huarong Yu

**Affiliations:** ^1^Department of Respiratory Medicine, The Second Affiliated Hospital of Chongqing Medical University, Chongqing, China; ^2^Graduate School, Chongqing Medical University, Chongqing, China; ^3^Stomatological Hospital of Chongqing Medical University, Chongqing, China; ^4^Department of Obstetrics and Gynaecology, The Second Affiliated Hospital of Chongqing Medical University, Chongqing, China

**Keywords:** clinical postgraduates, professional master’s, supervisors, curriculum, attitudes

## Abstract

**Background:**

Based on the recommendation of the Ministry of Education in China to differentiate between “academic” and “professional” degrees, medical schools offer both professional and academic degrees for postgraduates. In China, clinical postgraduates who are pursuing a professional master’s degree also participate in standardised residency training (SRT). However, little attention has been given to feedback from students and supervisors regarding postgraduate curricula.

**Methods:**

An online questionnaire was designed for clinical postgraduates with professional master’s degrees and their supervisors at Chongqing Medical University (CQMU), China. A total of 544 questionnaires from postgraduates and 220 questionnaires from supervisors were included for analysis.

**Results:**

Regarding the positive effect of public courses on professional research, 20.04% of clinical professional master’s degree students reported “a significant positive effect.” Compared with public courses, more postgraduates (33.46%) reported that professional courses had a “significant positive effect” on clinical work (*χ*^2^ = 25.00, *p < 0.05*). A total of 49.64% of respondents with clinical professional master’s degrees reported at least some repetition between postgraduate public courses and undergraduate courses. Of the postgraduates, 47.43% preferred online learning for public courses, whereas supervisors tended to prefer mixed online and offline learning. A total of 66.73% of postgraduates and 64.55% of supervisors suggested that public alternative courses should be offered to meet the needs of postgraduates. “Mental and health emotion management” and “employment and entrepreneurship guidance” were the public alternative courses that were most strongly preferred by both postgraduates and supervisors. With respect to improvements in self-knowledge and ability through different forms of professional learning, the responses of postgraduates and supervisors differed. According to postgraduates, the most effective type of learning was “participation in research projects,” whereas supervisors believed that “professional courses” were the most effective.

**Conclusion:**

There are differences between clinical postgraduates pursuing professional master’s degrees and their supervisors in terms of attitudes towards public and professional curricula. The results of this study may provide guidance to improve public and professional curricula for clinical professional master’s degree students.

## Introduction

Due to the increase in global life expectancy and population ageing, the demand for medical services and healthcare has increased significantly worldwide. In China, traditional postgraduate education in clinical medicine has prioritised the cultivation of academic skills, but the clinical proficiency of postgraduates in clinical medicine has failed to meet the increased demand for social healthcare (1). Due to the rapid development of medical services, healthcare, and higher education, China, which is the second most populous country in the world, has established a professional degree in clinical medicine to cultivate students’ applied medical skills, strong professional ability, and humanistic qualities, leading to better engagement in clinical work and healthcare (2). According to the recommendation of the Ministry of Education in China regarding the differentiation of “academic” and “professional” degrees, academic degree programmes aim to cultivate research-oriented personnel by training medical postgraduates in scientific research. In contrast, professional degree programmes aim to train individuals with outstanding skills who have received formal, high-level training in specialised technology (2, 3).

In China, postgraduates who pursue master’s degrees in clinical medicine need to complete postgraduate curricula and clinical rotations that last for 33 months (standardised residency training, SRT) and must write a dissertation (4, 5). Traditional SRT is designed for 5-year medical undergraduates with a bachelor’s degree followed by 3 years of residency training. Compared with traditional SRT, the combined training model for a clinical professional master’s degree requires postgraduates to develop skills in clinical research or literature reading to complete their dissertations. These programmes require students to have a strong learning ability to coordinate clinical rotations and scientific or clinical research plans. Therefore, the objective of curriculum construction for the clinical professional master’s degree should match the demands of clinical rotations, literature reading, and dissertation writing. Hence, most colleges in China have established online public alternative curricula to assist postgraduates who are pursuing professional master’s degrees by enhancing their performance in their research programmes (6).

In recent years, new designs for curricula for medical students have been developed, but nearly all of these reforms have focussed on undergraduate medical education. Clinical professionals in China rotate through different clinical departments for SRT, most of which are public or affiliated hospitals of medical universities (7). Hence, there are limited opportunities for centralised courses in professional clinical master’s programmes. Many medical universities, such as Chongqing Medical University (CQMU), offer most of their public courses for clinical professional masters online. There is limited information about the attitudes of professional postgraduates towards their curricula. Therefore, in recent years, reforms have focussed on curriculum design and reorganisation for clinical professional masters. Given this context, it is essential to understand clinical professional masters’ opinions of their courses, as well as their supervisors.

A large-scale survey was conducted by CQMU to investigate (1) the positive impact of courses on research, (2) the requirements for elective course content, and (3) the preferred organisation of curricula from the perspective of clinical professional masters and their supervisors. By investigating both supervisors and postgraduates, we intend to provide data for the future reforms of curricula for clinical professional master’s programmes in China.

## Methods

### Study design and data collection

An online questionnaire was designed for postgraduates and supervisors using the Questionnaire Star Platform, an anonymous online questionnaire tool. All questionnaires were administered and returned via the WeChat platform in April 2023. The questionnaire was administered for 10 days, followed by data collection and analysis. The questionnaire could be completed only once per IP address. Returned questionnaires with a total completion time of less than 5 min were considered invalid and were excluded from the final statistical analysis.

### Questionnaire and validity test

All the authors participated in developing the initial questionnaire based on a review of previous studies about surveys of curricula for postgraduate medical students (8–10). The questionnaire was modified during three rounds of Delphi consultation. Following the evaluation of the pilot survey among clinical professional masters and their supervisors at CQMU, the questionnaire was slightly modified based on expert consultation and sent to the WeChat platform (the English version is available in the Supplementary material). Two versions of the questionnaire were developed: one for postgraduates and one for supervisors. Each questionnaire included questions about the respondents’ basic information, public courses, and professional courses. Most items were single or multiple choice, i.e., respondents could select the most suitable answer or could select several options, respectively. In addition, a set of open-ended questions was included to collect feedback. The data were collected anonymously.

The questionnaire used three 4-point Likert scale questions to collect feedback from professional masters about the positive impact of the courses on their research (11). Finally, a validity test was performed based on the feedback of all the pilot surveys. The standardised Cronbach’s alpha coefficient was 0.874, the Kaiser–Meyer–Olkin value (12) was 0.717 (*p* = 0.000), and Bartlett’s test of sphericity was significant (chi-square = 324.055, *p* = 0.000).

### Participants

A total of 753 postgraduates who were pursuing clinical professional master’s degrees at CQMU from 2020 to 2022 received the online survey (student version) through WeChat, and 547 postgraduates provided their responses. We distributed the supervisor version of the online survey to 336 clinical professional postgraduate supervisors, and 226 provided online responses. Participants who failed to finish the questionnaire or who returned invalid questionnaires were excluded. Ultimately, a total of 544 questionnaires from postgraduates and 220 questionnaires from supervisors were included in our study. The details of the clinical professional postgraduates are listed in Table 1.

**Table 1:** Major characteristics of the enrolled clinical postgraduates with professional master’s degrees.

Characteristics		*N*	Percentage (%)
Grade	The first year grade	206	37.87
	The second year grade	197	36.21
	The third year grade	141	25.92
Gender	Female	312	57.35
	Male	232	42.65
Age	20–22 years	21	3.86
	23–25 years	274	50.37
	26–28 years	245	45.04
	Above 28 years	4	0.74
Major disciplines	Internal medicine	206	37.87
	Surgery	174	31.99
	Paediatrics	127	23.35
	Others	37	6.80

### Statistical analysis

The data were analysed with SPSS 17.0. Generally, categorical variables were compared between groups via Pearson’s chi-square test or Fisher’s exact test. Differences in ordinal variables were compared between groups via the Mann–Whitney *U*-test. *p*-values less than 0.05 were considered to indicate statistically significant differences.

## Results

Ultimately, 544 clinical professional master’s degree students and 220 clinical professional supervisors completed the questionnaire. As shown in Table 1, the ages of the postgraduates ranged from 23 to 28 years (95.41%), and the majority of the students specialised in internal medicine, surgery, or paediatrics (93.21%).

### Attitudes towards public curricula

**Figure 1:**
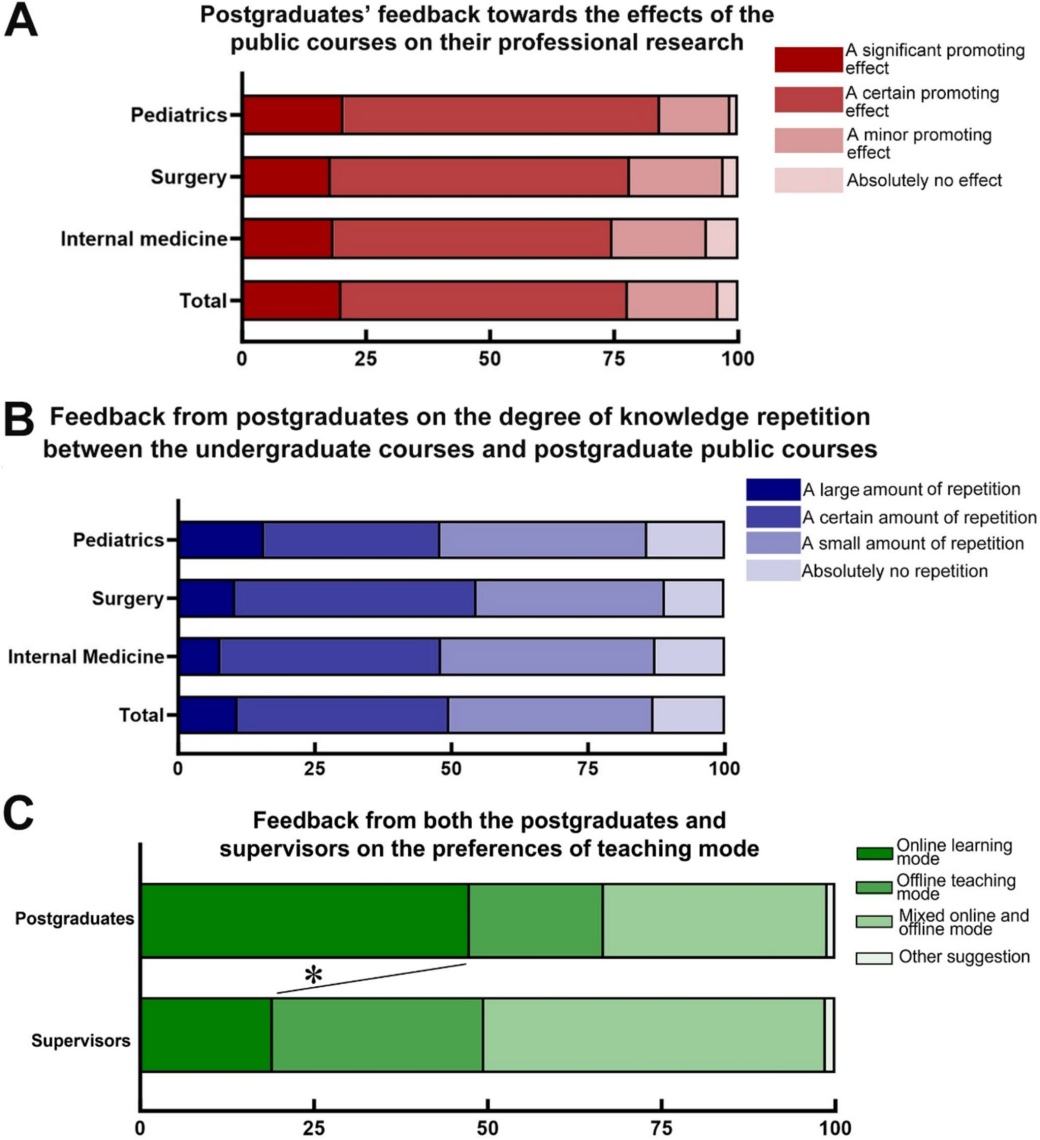
Postgraduates’ and supervisors’ attitudes towards professional curricula. **(A)** Feedback on the effects of public education on the professional research of postgraduates. **(B)** Postgraduates’ feedback on the degree of knowledge repetition between undergraduate courses and postgraduate public courses. **(C)** Feedback from postgraduates and supervisors on preferences for teaching mode. ^*^p < 0.05 between postgraduates and supervisors.

Regarding the question of “whether the public courses were helpful for professional research,” 20.04% and 57.72% of clinical professional masters reported “a significant promoting effect” and “a certain promoting effect,” respectively. However, 18.20% and 4.04% of the clinical professional masters reported “a minor promoting effect” and “absolutely no effect,” respectively (Figure 1A). Subgroup analysis revealed no significant differences in the responses among clinical professional masters based on their specialisation (i.e., internal medicine vs. surgery vs. paediatrics). Regarding the question about “the degree of knowledge repetition between undergraduate courses and postgraduate public courses,” 10.85% and 38.79% of clinical professional masters reported “a large amount of repetition” and “a certain amount of repetition,” respectively. Subgroup analysis indicated no significant differences among the students in terms of their speciality (i.e., internal medicine vs. surgery vs. paediatric medicine). Considering the limited number of postgraduates in fields other than internal medicine, surgery, and paediatrics, the subgroup analyses mentioned above were not included in the analysis (Figure 1B).

Questions about the teaching modes of public courses were administered to both postgraduates and supervisors. A total of 47.43% of postgraduates preferred an online learning mode for public courses, while only 19.3% of postgraduates preferred traditional offline teaching. A significant difference in preferences regarding the teaching mode was found between postgraduates and supervisors (*χ*^2^ = 56.47, *p < 0.05*). Supervisors tended to prefer online and offline mixed learning (49.09%) or traditional offline teaching (30.45%), whereas postgraduates preferred online learning (47.43%) (Figure 1C). A subgroup analysis was performed to identify differences across specialties (Table 2). Compared with postgraduates in internal medicine, surgical postgraduate students reported a stronger preference for online teaching (*p* < 0.05) (Table 2). There were no other significant differences in the public course preferences of postgraduates across different specialties (Table 2).

**Table 2:** Preferred modes of public courses among postgraduates and supervisors.

	Online teaching	Traditional classroom	Online and classroom mixed mode	Others
Postgraduates in internal medicine, *n* (%)	84 (40.78)	43 (20.87)	77 (37.38)	2 (0.97)
Postgraduates in surgery, *n* (%)	100 (57.47)^*^	28 (16.09)	43 (24.71)	3 (1.72)
Postgraduates in paediatrics, *n* (%)	55 (43.31)	27 (21.26)	44 (34.65)	1 (0.79)
Postgraduates in other disciplines, *n* (%)	19 (51.35)	7 (18.92)	11 (29.73)	0 (0.00)
Supervisors, *n* (%)	42 (19.09)	67 (30.45)	108 (49.09)	3 (1.36)

^*^p < 0.05 vs. “postgraduates in internal medicine”.

**Figure 2:**
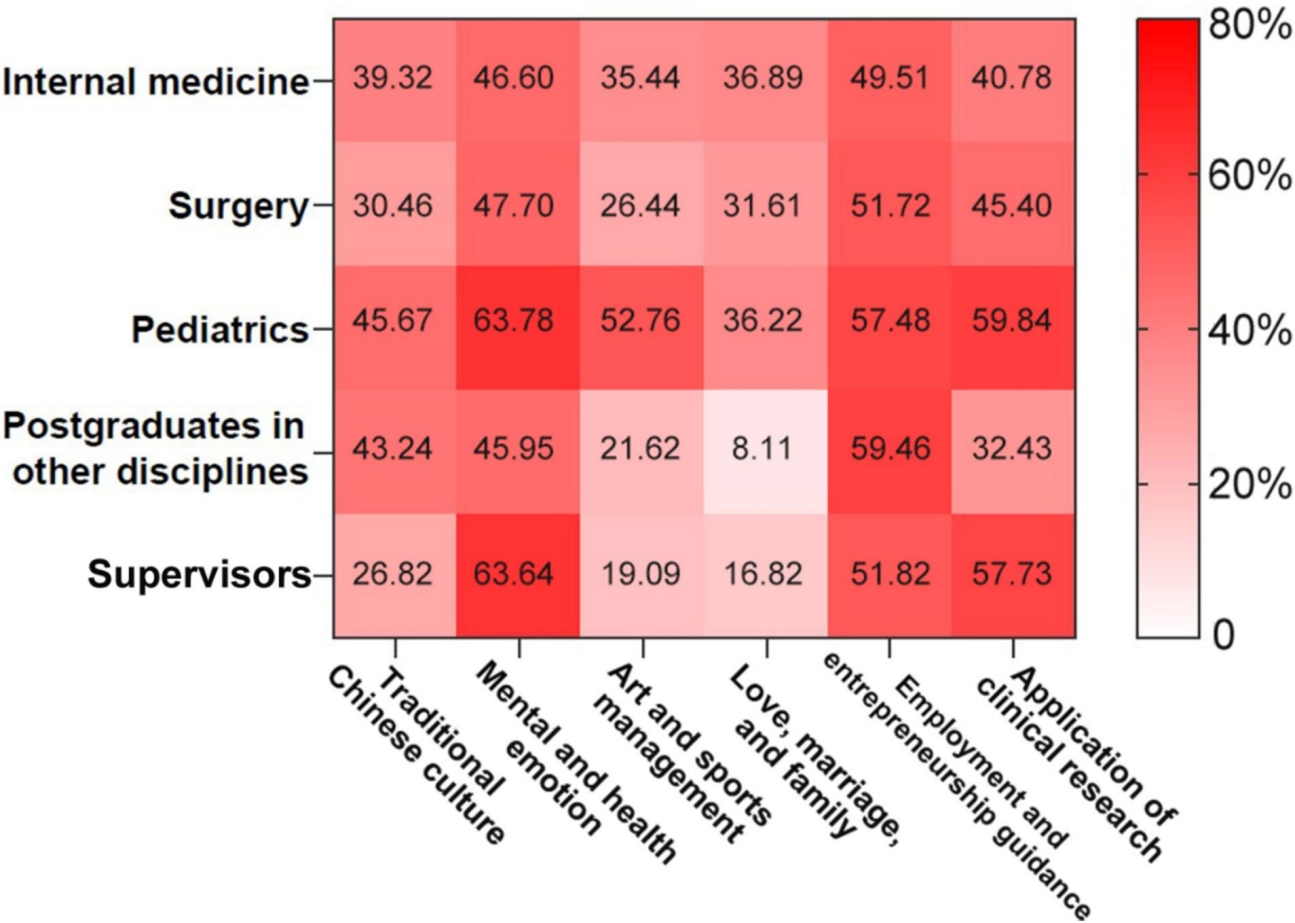
Heatmap for proposed additional topics of alternative public courses.

A total of 66.73% of postgraduates and 64.55% of supervisors suggested that additional public alternative courses should be offered to meet the needs of postgraduates. With respect to the new topics of these courses, postgraduates preferred courses about “mental and health emotion management,” “employment and entrepreneurship guidance,” and “application of clinical research.” These preferences were consistent with those of the supervisors (Figure 2).

### Attitudes towards professional curricula

**Figure 3:**
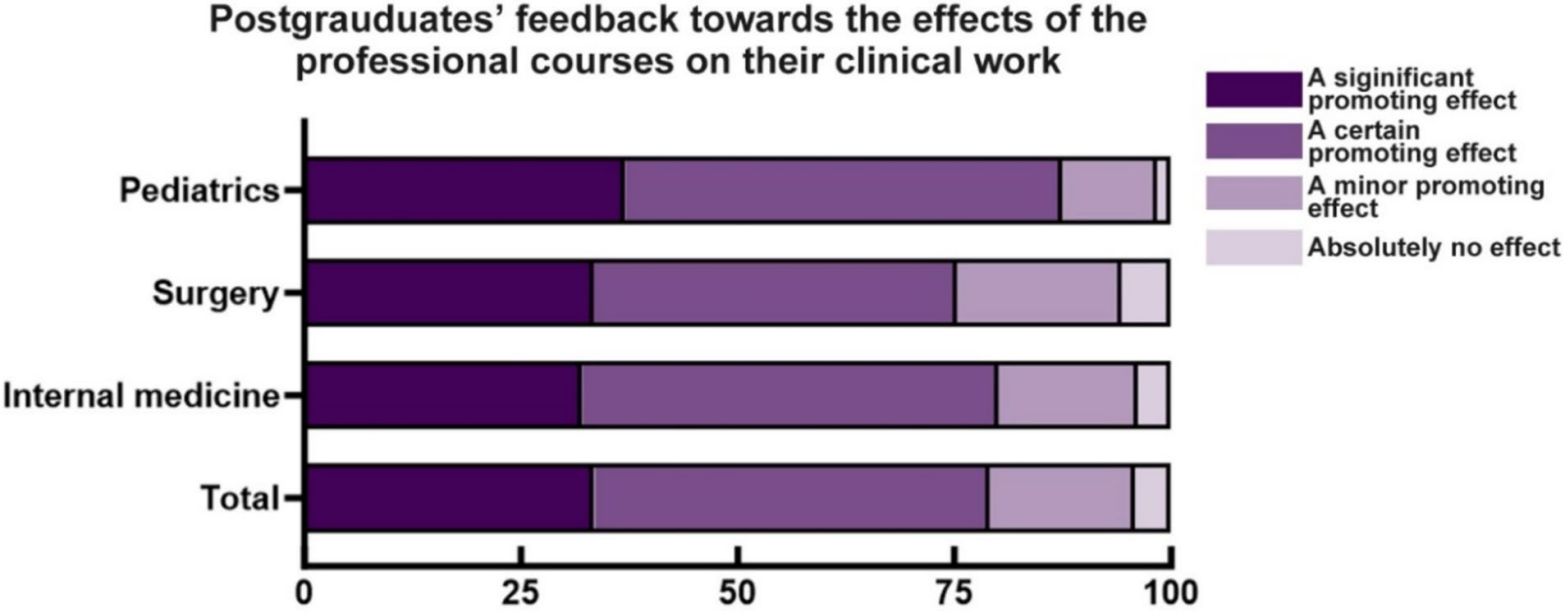
Postgraduates’ feedback on the effects of professional courses on their clinical work.

With respect to the question, “What do you prefer as the main type of professional course?,” 70.96% of postgraduates and 68.64% of supervisors preferred “mostly lectures accompanied by discussions.” Another question evaluated the positive effect of professional courses on the clinical work of postgraduates and found that 33.46% and 45.59% of postgraduates reported “a significant promoting effect” and “a certain promoting effect,” respectively. Compared with the “effect of public courses on research work” mentioned above, more postgraduates reported “a significant promoting effect” of professional courses on clinical work (*χ*^2^ = 25.00, *p* < 0.05) (Figure 3).

**Figure 4:**
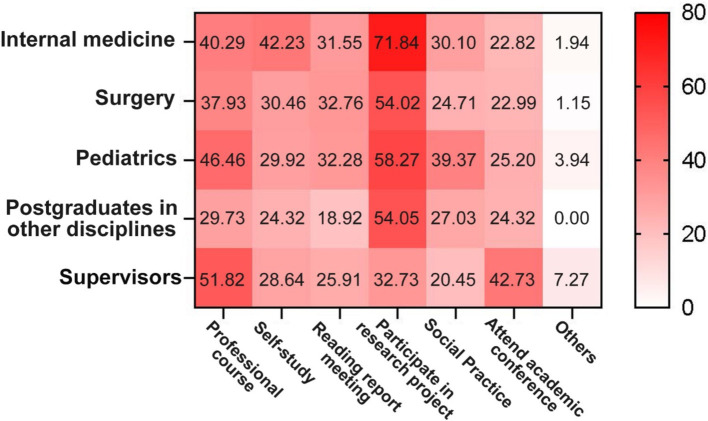
Heatmap for the efficacy of different forms of professional learning in improving knowledge and capabilities. Feedback was collected from both postgraduates and supervisors.

With respect to the improvement of self-knowledge and ability through different forms of professional learning, the most common preference among postgraduates was “participation in research projects” (61.76%), followed by “professional courses” (40.26%). A difference in preferences was observed between postgraduates and supervisors; the latter tended to prefer professional courses (51.82%) and academic conferences (42.73%) as highly efficient forms of learning (Figure 4).

## Discussion

In recent years, most medical colleges have paid less attention to postgraduate curricula than to undergraduate curricula. The underlying reason for this gap is that postgraduates have a certain degree of knowledge reserves. In the opinion of most educators, the main task of postgraduates is to complete research projects with their supervisors (13, 14). Currently, clinical postgraduates pursuing professional master’s degrees account for the majority of clinical master’s students in China (15). However, few studies have compared feedback from clinical professional master’s students and their supervisors with regard to postgraduate curricula.

In terms of the functional division of postgraduate curriculum design, public courses are designed for postgraduate research and dissertation preparation, while the professional curriculum places more emphasis on improving clinical thinking, professional skills, and abilities (16). One of the meaningful findings of this investigation is that compared with the positive impact of public courses on research, the positive impact of professional courses on clinical work is more significant according to the opinions of clinical postgraduate students. The answers to questions about whether there is a difference between the content of postgraduate public courses and the established knowledge system of undergraduate courses might partially explain this difference. In addition, compared to public courses, professional courses highly focus on specialised knowledge and clinical skill training, which complies with the SRT requirement of the clinical postgraduate (17). To address knowledge duplication in undergraduate courses, some medical colleges combine multiple forms of public curricula, such as traditional lectures, topic discussions, multidisciplinary seminars, scientific research training, and auxiliary teaching (16). In these public courses, clinical training and multidisciplinary seminars are the major components, which are quite different from the organisation of public courses at CQMU. With respect to the delivery of public courses, nearly half of the postgraduates preferred online courses, and surgical postgraduates were more inclined than internal medical postgraduates to select online courses. This phenomenon might be attributed to the time conflict between public courses and clinical work (18). In fact, to avoid conflict with clinical work, CQMU schedules the majority of public courses on weekends or after-work hours as much as possible. Individual semi-structured interviews with several postgraduates suggest that another major reason for the preference for online courses is that clinical postgraduates in different SRT hospitals must spend additional time travelling to colleges for public courses after work. Therefore, online public courses may be more acceptable for clinical master’s degree students. In contrast, fewer than 20% of the supervisors chose online teaching, and nearly half of them chose mixed online and offline learning approaches. Individual semi-structured interviews with several supervisors suggested that this disparity is mainly due to concerns about the effect of purely online courses. The combination of offline and online components can increase interaction with postgraduates and improve teaching quality (19, 20). Since the epidemic of COVID-19, online teaching has been widely used. For example, interactive online learning, which is derived from online teaching, and goal-oriented small private online courses (SPOCs), to some extent, have increased students’ interest in learning and the quality of online teaching (21). Therefore, diverse and thematic SPOCs may be a popular form of public courses for clinical professional students pursuing a master’s degree (22).

In terms of public alternative courses, “mental and health emotion management” and “employment and entrepreneurship guidance” have the highest level of demand among clinical postgraduates and supervisors due to pressure from the combined-training model, in which clinical postgraduates with professional master’s degrees need to complete SRT and postgraduate degree research within 3 years (23). Although the career direction of clinical professional postgraduates is clear, some of these students have difficulty in finding a satisfactory job. The underlying reason may be the dual structure of urban and rural areas in China, which results in an urban–rural split in terms of economics, education, and medical care. Furthermore, few clinical professional masters select rural medical institutions or primary medical institutions as their first choice after graduation (24). In addition, insufficient opportunities, limited availability of training positions, and the lack of adequate teaching by supervisors during SRT are the possible explanations for difficulty in finding a satisfactory job, to some extent (17). Therefore, problem-oriented micro-lectures associated with psychological counselling and employment guidance are needed.

Another important finding of this investigation is that, from the perspective of postgraduates, the most efficient method for improving their self-knowledge and capability is to “participate in research projects.” Supervisors are more concerned with improving the professional ability of postgraduates through professional lectures and initiatives in academic conferences and discussions. As a goal-or outcome-oriented learning mode, participating in clinical research is an efficient way to improve the professional capability of clinical postgraduates. Traditionally, clinical postgraduates’ participation in clinical research should be preceded by the completion of theoretical courses or lectures to obtain sufficient knowledge and pass topic selection. From the perspective of goal-or outcome-oriented learning, early experience in a research programme with guidance from a supervisor might also be an efficient learning mode (25), especially for clinical postgraduates pursuing professional master’s degrees who spend the majority of their time in SRT. Further research on clinical postgraduates with professional master’s degrees may focus on the design of outcome-oriented professional micro-lectures in which supervisors participate. Encouraging early participation in and conducting clinical research may be a suitable method to effectively improve the professional skills of clinical postgraduates.

Our study comprehensively surveyed the attitudes of clinical professional masters and their supervisors towards the curriculum. However, this study has several limitations. First, questionnaires were not recovered from all clinical professional masters at CQMU. Second, the postgraduates surveyed in this study experienced the COVID-19 pandemic. During the COVID-19 pandemic, several courses have to be performed online, which may have potentially influenced the postgraduates’ attitude towards the curriculum. Third, the survey was administered at CQMU, a single centre in China. Multicentre surveys with larger samples may be necessary to further verify our conclusions.

## Conclusion

The major conclusions of our survey are as follows. (1) There are several differences between clinical postgraduates pursuing professional master’s degrees and their supervisors in terms of their attitudes towards public and professional curricula. Diverse and thematic small online courses might be popular among clinical masters. (2) “Mental and health emotion management” and “employment and entrepreneurship guidance” are the two most preferred alternative courses among both clinical postgraduates and supervisors. (3) Encouraging early participation in and conducting a moderate amount of clinical research may effectively improve the professional skills of clinical postgraduates.

## Data Availability

The raw data supporting the conclusions of this article will be made available by the authors, without undue reservation.
