# Supplementary material for: A survey of attitudes towards the curriculum for clinical medicine postgraduates pursuing professional master’s degrees: perspectives of supervisors and students
Source: Front Med (Lausanne). 2024 Dec 10;11:1488139. doi: 10.3389/fmed.2024.1488139 (PMC11666361; doi:10.3389/fmed.2024.1488139)
Supplement: Supplementary file 1 [file Data_Sheet_1.PDF]

Supplementary Material

Questionnaire on attitudes towards the curriculum for clinical medicine postgraduates pursuing professional master's degrees (Student Edition)

Part1. Basic information

1. Which grade are you in now?

○First year grade

○Second year grade

○Third year grade

2. Your gender is

○Female

○Male

3. Which discipline is you specialized in?

○Internal Medicine

○Surgery

○Pediatrics

○Others(ex. Obstetrics and Gynecology, Ophthalmology, Otolaryngology et.al)

Part2. Public curricula

4. Whether the public courses were helpful for professional research

○A significant promoting effect

○A certain promoting effect

○A minor promoting effect

○Absolutely no effect

5. The degree of knowledge repetition between the undergraduate courses and postgraduate public courses.

○A large amount of repetition

○A certain amount of repetition

○A small account of repetition

○Absolutely no repetition

6. Which teaching mode do you prefer as the major manner for the public courses.

○Online learning mode

○Traditional offline mode

○Mixed online and offline mode

○Others____________________

7. Do you think additional public alternative courses needed be added?

○Yes

○No

8. Which one or more new topics of public alternative courses do you think should be added? [Multiple choices]

○Traditional Chinese Culture

○Mental and health Emotion management

○Art and Sports

○Love, Marriage and Family

○Employment and Entrepreneurship Guidance

○Application of clinical research

Part3. Professional curricula

9. Which manner do you prefer as the major form for professional courses

○Just lecture

○Mostly lecture, accompanied by discussion

○Mostly discussion, accompanied by lecture

○Just discussion

10. Whether the professional courses were helpful for clinical work of postgraduates

○A significant promoting effect

○A certain promoting effect

○A minor promoting effect

○Absolutely no effect

11. Among the following forms of professional learning, the highly effective way to improve

self-knowledge and capability are___.   [Multiple choices]

○Professional course

○Self-study

○Reading report meeting

○Participate in research project

○Social practice

○Attend academic conferences

○Others

Questionnaire on attitudes towards the curriculum for clinical medicine postgraduates pursuing professional master's degrees (Supervisor Edition)

Part1. Basic information

1. Your supervisor's role is____

○Master's supervisor

○Doctor's supervisor

Part2. Public curricula

2. Which teaching mode do you prefer as the major manner for the postgraduates' public courses.

○Online learning mode

○Traditional offline mode

○Mixed online and offline mode

○Others____________________

3. Do you think additional public alternative courses needed be added?

○Yes

○No

4. Which one or more new topics of public alternative courses do you think should be added? [Multiple choices]

○Traditional Chinese Culture

○Mental and health Emotion management

○Art and Sports

○Love, Marriage and Family

○Employment and Entrepreneurship Guidance

○Application of clinical research

Part3. Professional curricula

5. Which manner do you prefer as the major form for professional courses

○Just lecture

○Mostly lecture, accompanied by discussion

○Mostly discussion, accompanied by lecture

○Just discussion

6. Among the following forms of professional learning, in your opinion, the highly effective way to improve the knowledge and capability of the clinical postgraduates are___. [Multiple choices]

○Professional course

○Self-study

○Reading report meeting

○Participate in research project

○Social practice

○Attend academic conferences

○Others
